# 16S rRNA Datasets of Gut Microbiota in Stent-Based Diverting Technique and Ileostomy from A Prospective, Open-Label, Multicenter Randomized Controlled Trial

**DOI:** 10.1038/s41597-026-08247-3

**Published:** 2026-09-30

**Authors:** Zhongyu Wu, Yifan Tong, Weiping Chen, Wenbin Chen, Lingfei Li, Diyu Huang, Zheyong Li, Zhangfa Song, Xiujun Cai

**Affiliations:** 1 Department of Surgical Oncology, Sir Run Run Shaw Hospital, School of Medicine, Zhejiang University, Hangzhou, China; 2 Department of General Surgery, Sir Run Run Shaw Hospital, School of Medicine, Zhejiang University, Hangzhou, China; 3 Zhejiang Key Laboratory of Precise Diagnosis and Treatment of Abdominal Infection, Hangzhou, China; 4 Department of Colorectal Surgery, The First Affiliated Hospital, School of Medicine, Zhejiang University, Hangzhou, China; 5 Department of Colorectal Surgery, Zhejiang Cancer Hospital, Hangzhou, China; 6 National Engineering Center for Minimally invasive Devices and Innovative Applications, Sir Run Run Shaw Hospital, School of Medicine, Zhejiang University, Hangzhou, China; 7 Department of General Surgery, Alaer Hospital, School of Medicine, Tarim University, Alar, China; 8 Department of Colorectal Surgery, Sir Run Run Shaw Hospital, School of Medicine, Zhejiang University, Hangzhou, China

## Abstract

Accumulating evidence implicates gut dysbiosis in postoperative complications and delayed intestinal recovery after rectal cancer surgery. Although stent-based diverting technique (SDT) and conventional ileostomy exert divergent impacts on gut microbial balance, high-quality comparative microbiome data from randomized trials are lacking. We report a 16S rRNA amplicon dataset from 80 patients randomized to SDT (n = 37) or ileostomy (n = 43), with fecal samples collected preoperatively and on postoperative day 90. We characterized phylum-level taxonomic composition, α-diversity and intergroup β-diversity across four subgroups, and identified discriminatory taxa via LEfSe. Functional prediction detected differential metabolic pathways after surgery. We additionally performed correlation analyses to link clinical covariates with microbial signatures. This public dataset enables independent reprocessing and cross-study comparisons, facilitating exploration of diversion-strategy-specific microbial remodeling and validation of SDT’s gut microbiota-preserving capacity.

## Background & Summary

Anastomotic leakage constitutes a severe complication following rectal cancer surgery, which poses a substantial threat to patients’ health^1,2^. Ileostomy is the most widely adopted protective intervention worldwide, yet this procedure leads to stoma-related complications requiring secondary reversal surgery together with gut microbiota dysbiosis^3^. Ileostomy interrupts intestinal continuity, forming a functionally isolated distal bowel segment accompanied by severe gut microbiota dysbiosis. Prior studies have further confirmed that such dysbiosis contributes to the pathogenesis of diversion colitis (DC) and is linked to poor oncological outcomes^4–6^. In normal intestines, commensal bacteria generate short-chain fatty acids to maintain mucosal integrity. After fecal diversion, lack of luminal contents suppresses beneficial microbes and facilitates the expansion of pathogenic bacteria, whose metabolites trigger intestinal inflammation^7–16^.

The stent-based diverting technique (SDT) represents a novel stoma-free approach developed by our team for protecting low rectal anastomoses^7^. In contrast to conventional ileostomy, SDT markedly shortens the duration of intestinal diversion. Normal bowel function is generally restored within 3 to 4 weeks, which accelerates the rapid recovery of gut microbiota. In our previous trial, we demonstrated that SDT, when compared with ileostomy, shortened hospital length of stay, reduced medical expenses, and improved patients’ quality of life^8^. The prior work focused exclusively on clinical outcomes, did not include any gut microbiota-related data or analyses. However, changes in gut microbiota following this procedure remain uncharacterized, and the beneficial effects of SDT on gut microbial homeostasis have not yet been elucidated.

The gut microbiota sub-study was prespecified in the original trial protocol^8^. Using fecal samples collected from the same trial cohort at preoperative baseline and 90 days after surgery, we performed 16S rRNA gene sequencing and standardized bioinformatics processing. All microbiota-related data contained in this work are previously unpublished, and constitute a new, independent data resource generated from the established clinical trial cohort. We systematically analyzed microbial diversity, community structure, intergroup differences, functional profiles, and microbe-environment associations. This newly released dataset reveals the divergent impacts of these two diversion procedures on gut microbial homeostasis. Our findings may lay the groundwork for targeted interventions to reverse ileostomy-related gut dysbiosis and promote postoperative recovery.

## Methods

### Study design and ethical statement

Three hospitals in China participated in this randomized, multicenter, open-label trial from December 2021 to March 2023 in accordance with the Declaration of Helsinki. The complete study protocol and preliminary clinical outcomes of this trial were reported in our previously published study^9^. This retrospective study was conducted using de-identified clinical and 16S rRNA sequencing data, and was approved by the Ethics Committee of Sir Run Run Shaw Hospital, Zhejiang University (SRRSH-2026-Research-2178). A waiver of informed consent was granted by the ethics committee for non-identifying data collected retrospectively.

In this trial, 80 patients with rectal cancer were randomized to the SDT group (n = 37) or the ileostomy (ILE) group (n = 43). Two patients initially assigned to the SDT group underwent conversion to ileostomy. Accordingly, the as-treated population comprised 35 patients in the SDT group and 45 patients in the ILE group.

### Human feces sample collection

**Fig. 1:**
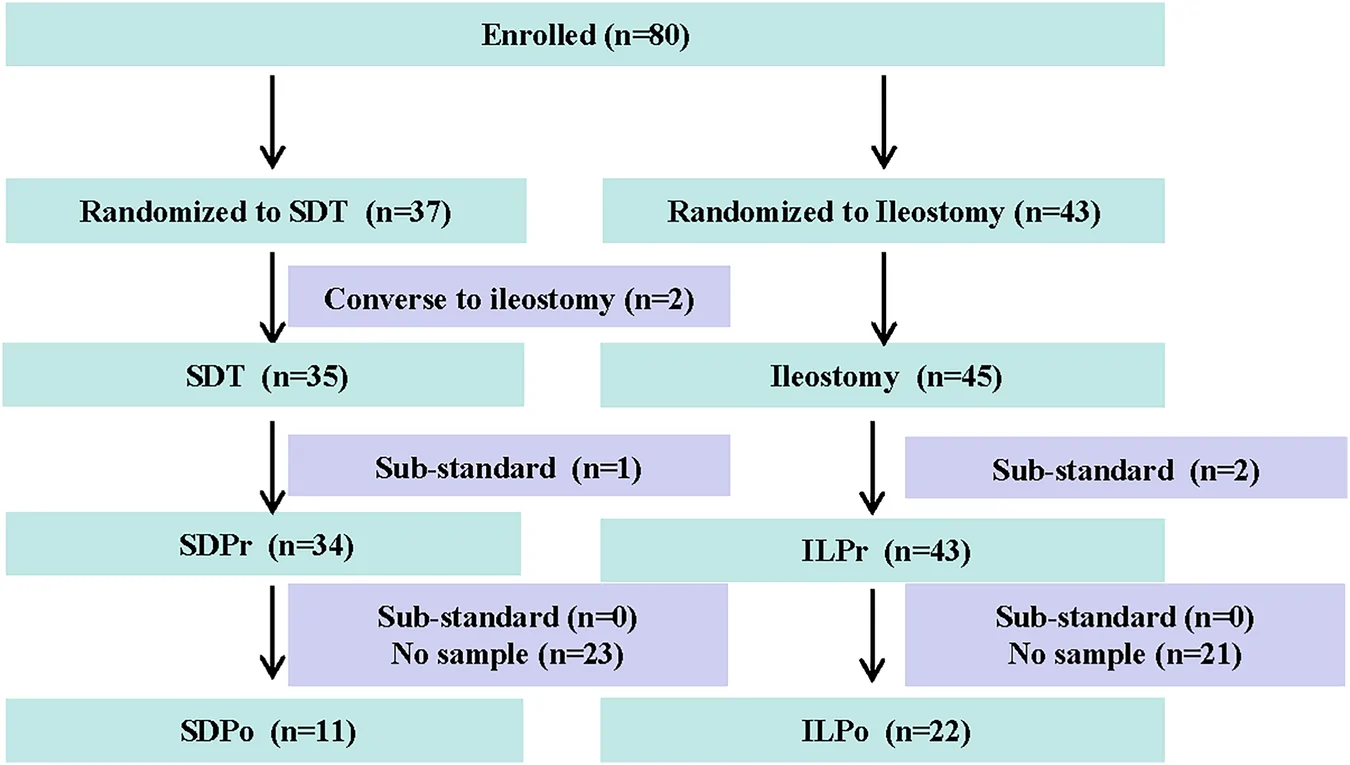
Flowchart of this trial. Samples were coded as SDPr (preoperative SDT), SDPo (postoperative SDT), ILPr (preoperative ILE), and ILPo (postoperative ILE). SDT, Stent-Based Diverting Technique.

Fecal samples were collected preoperatively and at postoperative day 90 with sterile cotton swabs from the deep rectum, about 5–7 cm proximal to the anal verge. The total initial collection contained 113 specimens. After quality control, 3 invalid samples were excluded. The final dataset included 34 preoperative and 11 postoperative samples from the SDT group, alongside 43 preoperative and 22 postoperative samples from the ILE group (Fig. 1). Baseline demographic and clinical characteristics are summarized in Table 1.

**Table 1:** Characteristics of patients.

	Ileostomy (n = 43)	SDT (n = 34)	P value
Age, y	59.1 ± 9.5	64.6 ± 8.3	0.008
BMI, kg/m^2^	23.4 ± 2.7	25.0 ± 4.2	0.059
Gender, (%)			0.515
Female	17 (39.5)	11 (32.4)	
Male	26 (60.5)	23 (67.6)	
ASA, (%)			0.091
I	14 (32.6)	6 (17.6)	
II	29 (67.4)	26 (76.5)	
III	0 (0)	2 (5.9)	
Hypertension, (%)			0.139
No	31 (72.1)	19 (55.9)	
Yes	12 (27.9)	15 (44.1)	
Diabetes, (%)			0.745
No	33 (76.7)	25 (73.5)	
Yes	10 (23.3)	9 (26.5)	
Heart Disease, (%)			0.192
No	43 (100)	32 (94.1)	
Yes	0 (0)	2 (5.9)	
Respiratory Disease, (%)			1.000
No	40 (93.0)	32 (94.1)	
Yes	3 (7.0)	2 (5.9)	
Neoadjuvant Therapy, (%)			0.146
No	34 (79.1)	31 (91.2)	
Yes	9 (20.9)	3 (8.8)	
Differentiation, (%)			0.417
Unreported	2 (4.7)	1 (2.9)	
Low	12 (27.9)	5 (14.7)	
Middle	25 (58.1)	26 (76.5)	
High	4 (9.3)	2 (5.9)	
Pathology, (%)			1.000
Adenocarcinoma	41 (95.3)	34 (100.00)	
Mucinous adenocarcinoma	1 (2.3)	0 (0.00)	
Neuroendocrine tumor	1 (2.3)	0 (0.00)	
T stage, (%)			0.380
1	5 (11.6)	8 (23.5)	
2	7 (16.3)	4 (11.8)	
3	25 (58.1)	15 (44.1)	
4	6 (14.0)	7 (20.6)	
N stage, (%)			0.562
0	23 (53.49)	14 (41.18)	
1	14 (32.56)	14 (41.18)	
2	6 (13.95)	6 (17.65)	

Data are presented as mean with standard deviation or numbers with percentages.

SDT, Stent-Based Diverting Technique; BMI, body mass index (calculated as weight in kilograms divided by height in meters squared); ASA, American Society of Anesthesiologists; TNM stage was according to the American Joint Committee on Cancer (8th version).

### Sequencing

The basic experimental workflow of microbial DNA extraction and 16S rRNA sequencing followed the mature methods reported in previous literature^17^. 16S rRNA genes of distinct regions (16SV3-V4) were amplified used specific primer with the barcode. All PCR reactions were carried out with 15 µL of Phusion® High-Fidelity PCR Master Mix (New England Biolabs); 0.2 µM of forward and reverse primers, and about 10 ng template DNA. Thermal cycling consisted of initial denaturation at 98°C for 1 min, followed by 30 cycles of denaturation at 98°C for 10 s, annealing at 50°C for 30 s, and elongation at 72°C for 30 s and 72°C for 5 min. According to the platform guidelines, this temperature can produce qualified amplicons with few non-specific bands when the template is precisely quantified and PCR cycles are well controlled. The PCR products were purified using magnetic bead purification. Samples were mixed in equidensity ratios based on the concentration of PCR products. After thorough mixing, the PCR products were detected and target bands were recovered. Sequencing libraries were generated and indexes were added. The library was checked with Qubit and real-time PCR for quantification and bioanalyzer for size distribution detection. Quantified libraries were pooled and sequenced on Illumina platforms, according to effective library concentration and data amount required.

## Data Record

In the present study, all raw 16S rRNA gene amplicon sequencing data generated in this study were submitted to the NCBI Sequence Read Archive (SRA) under accession SRP711022^18^. The complete dataset is accessible via BioProject accession PRJNA1480144, which corresponds to the SRA study ID SRP711022, and contains paired-end sequencing reads from 110 fecal specimens collected at preoperative baseline and postoperative day 90 from patients in the SDT and ileostomy cohorts. Processed data were uploaded to the NCBI Gene Expression Omnibus (GEO) with accession number GSE336197^19^. All visualized analytical outputs are provided in the Data Overview section of this manuscript.

## Data Overview

**Fig. 2:**
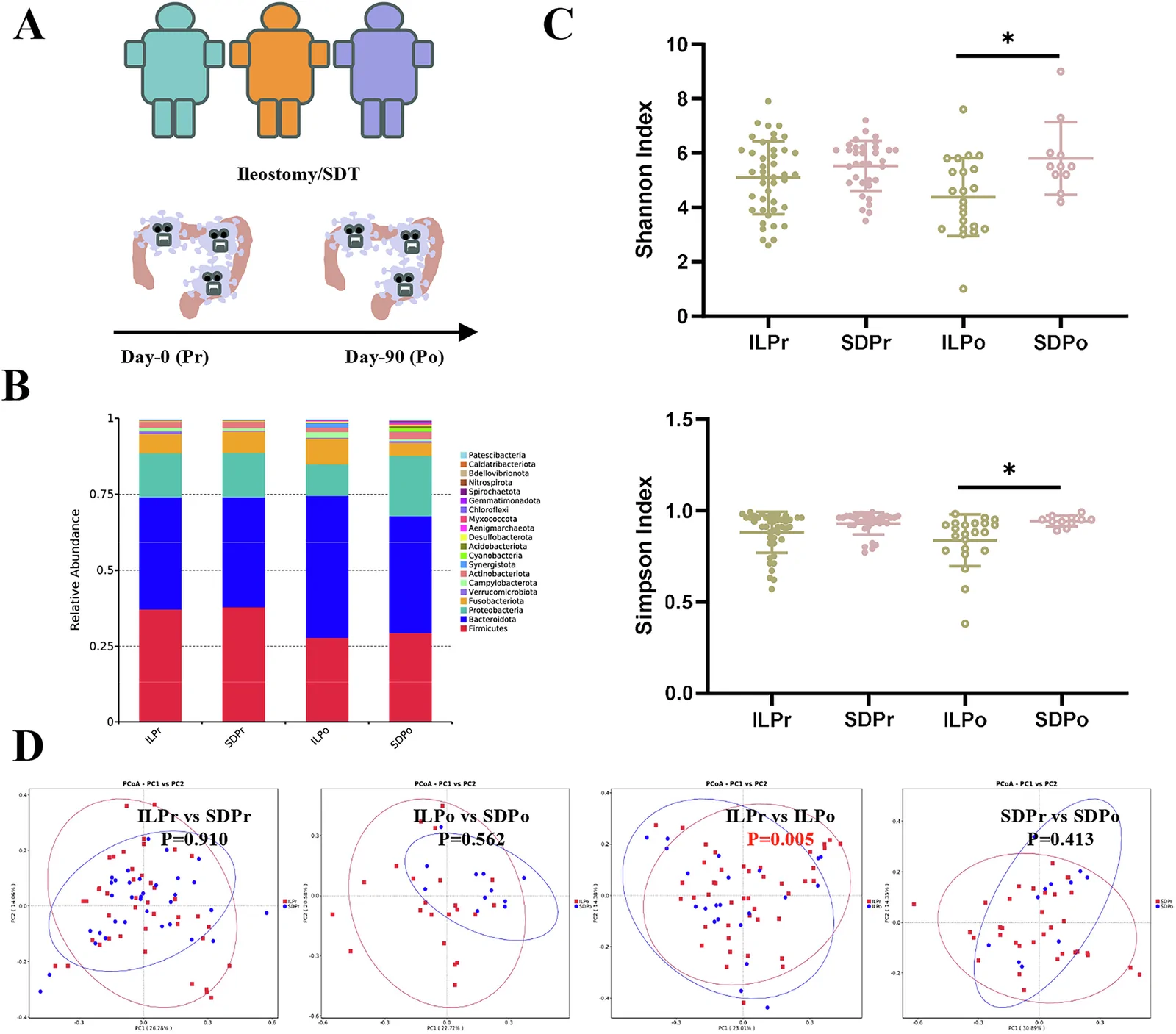
Composition and diversity. (**A**) Trial procedures: Fecal samples were collected from patients preoperatively and at 90 days postoperatively. (**B**) Gut microbiota composition at the phylum level across all groups. (**C**) Alpha diversity including Shannon and Simpson indices across all groups. (**D**) PCoA analysis revealed preoperative and postoperative differences in gut microbiota among the groups, and Adonis test verified the statistical significance of these β-diversity variations. “*” represented the P value less than 0.05.

**Fig. 3:**
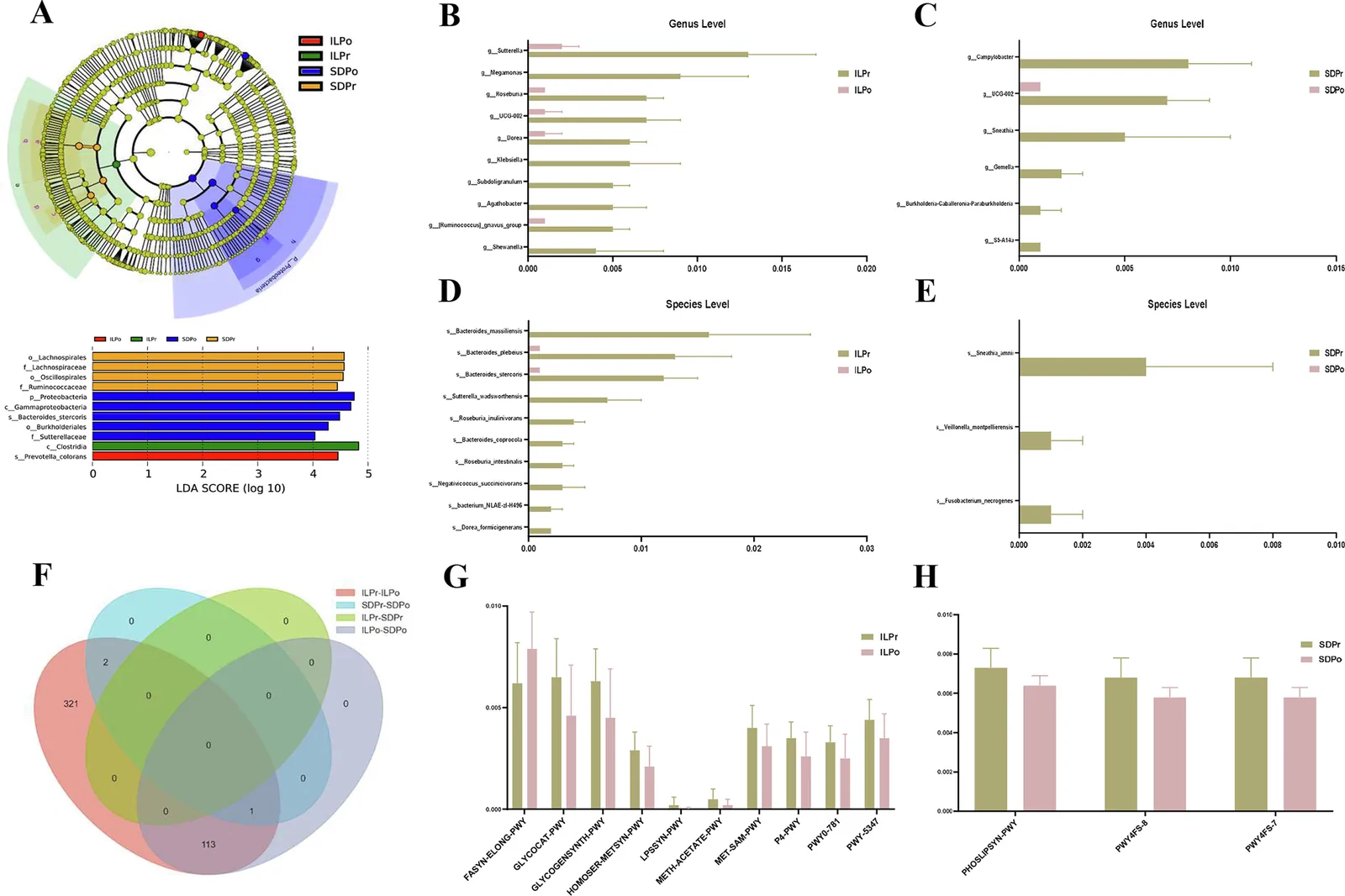
Differential analysis and functional prediction. (**A**) Differential analysis by LEfSe analysis across all group, the threshold LDA score is 4. (**B**) Representative microbiota in the metagenome differential analysis between preoperative and postoperative ileostomy groups at the genus level. (**C**) Representative microbiota in the metagenome differential analysis between preoperative and postoperative ileostomy groups at the species level. (**D**) Representative microbiota in the metagenome differential analysis between preoperative and postoperative SDT groups at the genus level. (**E**) Representative microbiota in the metagenome differential analysis between preoperative and postoperative SDT groups at the species level. (**F**) Functional prediction analysis across all groups. (**G**) Representative pathway enriched between preoperative and postoperative ileostomy groups. (**H**) Three pathway enriched between preoperative and postoperative SDT groups.

**Fig. 4:**
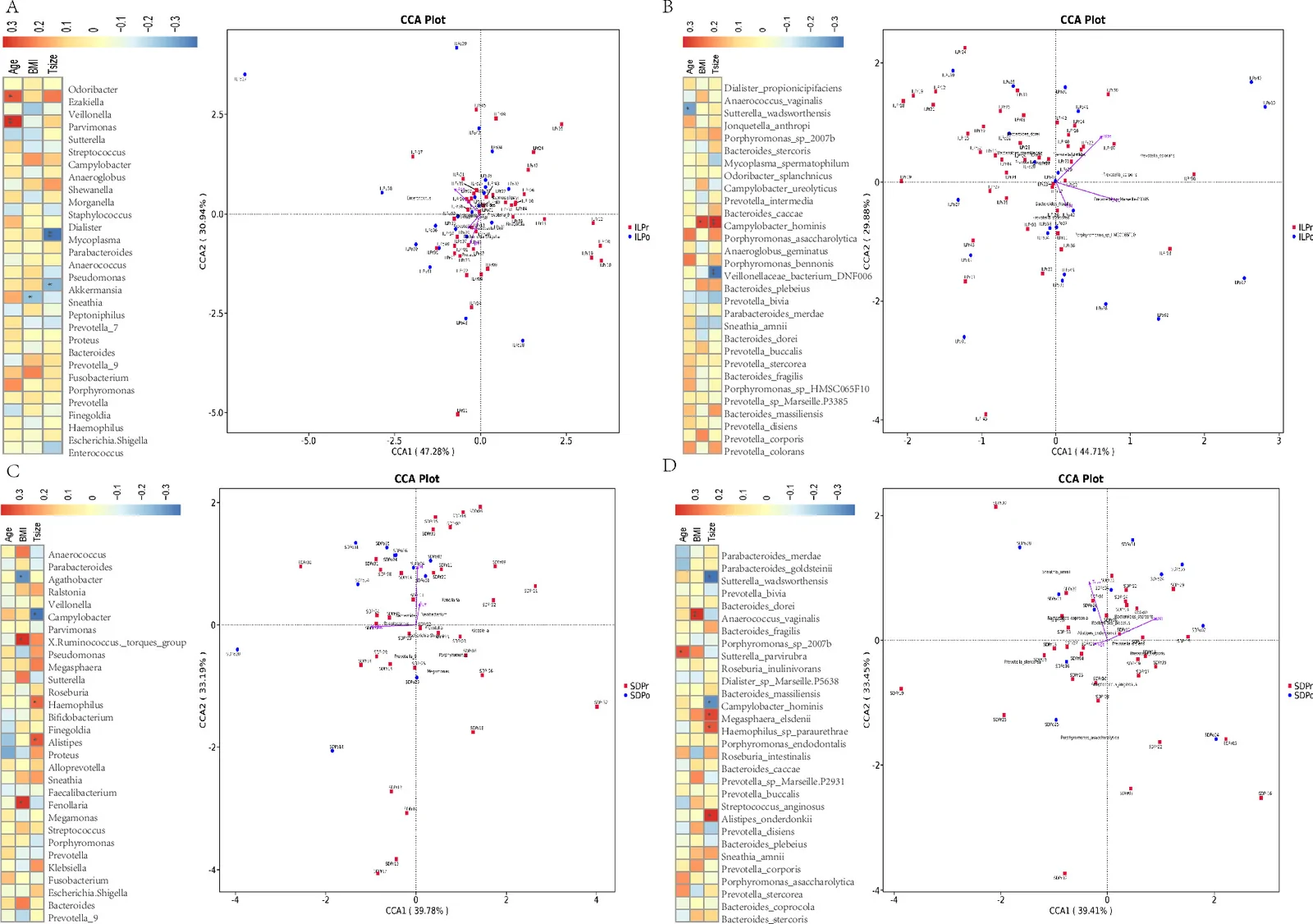
Canonical correspondence analysis. (**A**) Canonical correspondence analysis (CCA) of environment factors (age, BMI, tumor size) between preoperative and postoperative ileostomy groups at genus level. (**B**) CCA of environment factors between preoperative and postoperative ileostomy groups at species level. (**C**) CCA of environment factors between preoperative and postoperative SDT groups at genus level. (**D**) CCA of environment factors between preoperative and postoperative SDT groups at species level. “*” represented the P value less than 0.05, and “**” represented the P value less than 0.01. BMI, body mass index. Tsize, tumor size.

For clear subgroup differentiation, preoperative and postoperative specimens from the SDT cohort were labeled SDPr and SDPo, whereas samples from the ileostomy (ILE) group were coded ILPr and ILPo (Fig. 2A). Figure 2B–D summarizes phylum-level community composition, alpha diversity (Shannon and Simpson indices), and cross-group beta-diversity dissimilarities across the four subgroups. LEfSe analysis identified robust taxonomic enrichment patterns distinguishing these cohorts (Fig. 3A). Metagenomic differential profiling further showed significantly reduced bacterial species richness in postoperative samples relative to matched preoperative specimens for both surgical arms: ILPo versus ILPr, and SDPo versus SDPr. Taxa with markedly depleted abundances are visualized in Fig. 3B–E. We next performed functional prediction to characterize biological perturbations driven by microbial dysbiosis. The ILPo group exhibited the broadest spectrum of enriched functional pathways compared with ILPr (Fig. 3F). No differential pathways were detected at baseline between groups, yet 113 distinct pathways emerged postoperatively. Intragroup comparisons further uncovered 437 altered pathways between pre- and post-operative time points within the ileostomy cohort (Fig. 3G,H). Given the critical role of clinical covariates in shaping gut microbial profiles and host-microbe crosstalk, we carried out comprehensive correlation analyses linking clinical metadata and taxonomic abundances (Fig. 4). Remaining supporting data are compiled in Supplementary File 1.

## Technical Validation

Strict adherence to the participant inclusion and exclusion criteria was maintained throughout the study. Raw sequencing data were first demultiplexed based on barcodes to assign sequences to individual samples, after which barcodes and primer sequences were trimmed from all reads. The paired-end R1 and R2 reads were then assembled using the FLASH software. The assembled Tags underwent quality control to produce Clean Tags. Finally, chimeric sequences were filtered out, and the resulting valid sequences were retained as Effective Tags for all downstream analyses.

Raw paired-end sequencing reads were first demultiplexed into individual samples based on unique barcodes, and barcode and primer sequences were trimmed from all raw reads; the qualified reads were then assembled into raw tags using the open-source FLASH tool (v1.2.11), which was designed for merging overlapping paired-end short reads derived from identical DNA fragments, and the resulting raw tags were processed with strict quality filtering via fastp (v0.23.1) to remove low-quality sequences and generate high-quality clean tags. Subsequently, chimeric sequences present in the clean tags were identified and removed using the package (v2.16.0) through sequence alignment against the authoritative SILVA 16S/18S rRNA database and UNITE ITS database, and the final valid effective tags obtained after comprehensive filtering were adopted for all subsequent bioinformatics analyses.

For the Effective Tags obtained previously, denoise was performed with DADA2 or deblur module in the QIIME2 software to obtain initial ASVs (Amplicon Sequence Variants). Species annotation was performed using QIIME2 software. The annotation database is Silva Database, while for ITS, it is Unite Database. To ensure sufficient sequencing depth, rarefaction curves based on ASV richness were generated for all samples. The curves plateaued at the current sequencing depth, indicating that the majority of microbial diversity was captured.

This study has several limitations. First, 16S rRNA gene sequencing only enables taxonomic identification at the genus and species levels, and microbial functional profiles in this work were merely predicted rather than experimentally verified. Second, fecal samples were only collected at a single postoperative time point, failing to capture the long-term dynamic alterations of gut microbiota. Third, the postoperative sample size of the SDT group was relatively small (n = 11), which may compromise the statistical reliability of the results. Further large-scale randomized controlled trials are therefore required to elaborate the underlying mechanisms.

## Supplementary information

Supplementary Information

Supplementary Information

## Data Availability

No custom code was used. All analyses were performed as follows: R (version 4.4.0); Python version 3.6.13; Cutadapt version 3.3; Flash version 1.2.11; Fastp version 0.23.1; Vsearch version 2.16.0; Qiime2 version 202202; Perl version 5.26.2; Lefse version 1.1.01; Picrust2 version 2.3.0.
