# Supplementary material for: 16S rRNA Datasets of Gut Microbiota in Stent-Based Diverting Technique and Ileostomy from A Prospective, Open-Label, Multicenter Randomized Controlled Trial
Source: Sci Data. 2026 Sep 30;13:1366. doi: 10.1038/s41597-026-08247-3 (PMC13627693; doi:10.1038/s41597-026-08247-3)
Supplement: Supplementary file 1 [file 41597_2026_8247_MOESM1_ESM.pdf]

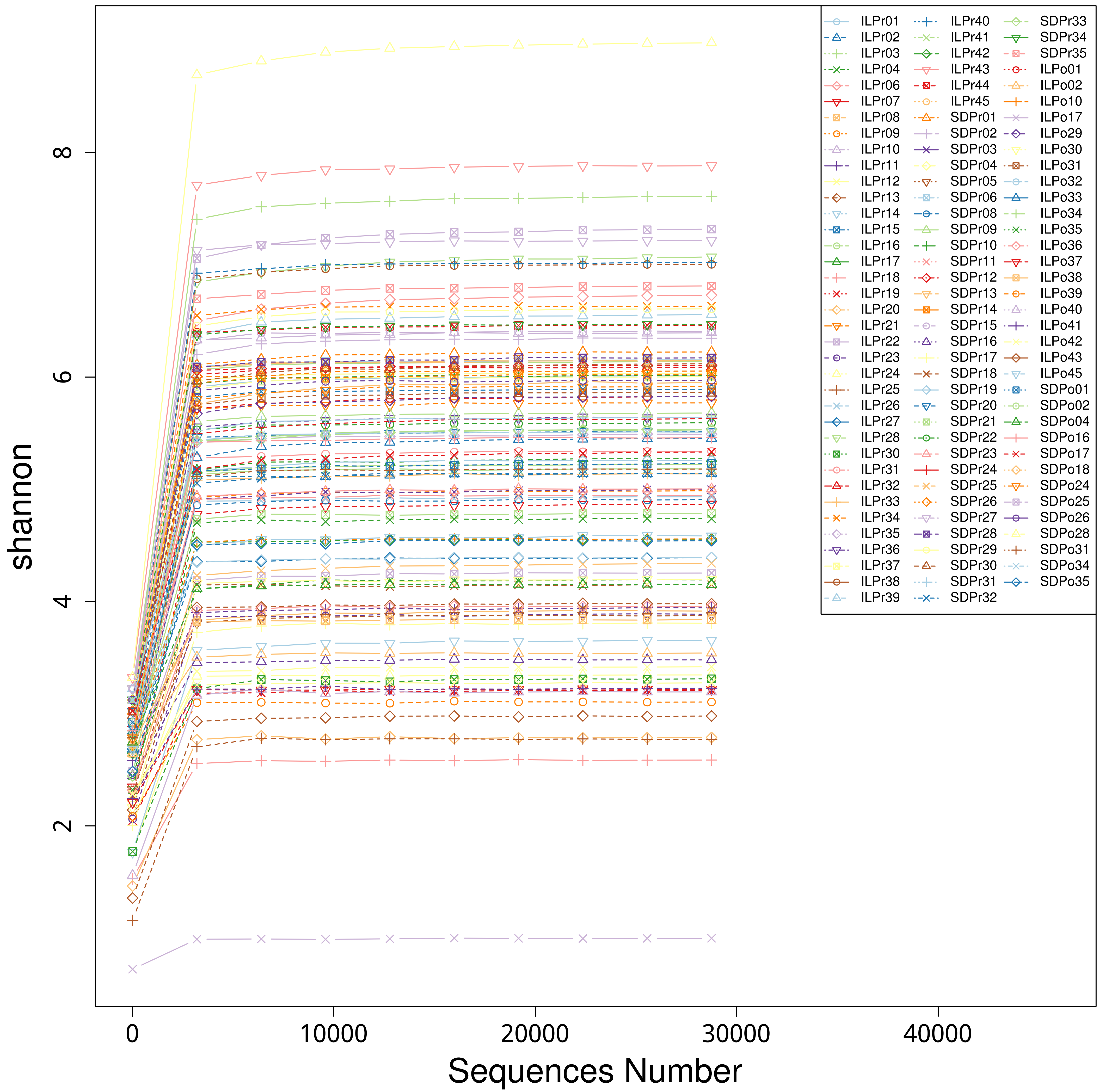
shannon
Sequences Number
2
4
6
8
0
10000
20000
30000
40000
ILPr01
ILPr02
ILPr03
ILPr04
ILPr06
ILPr07
ILPr08
ILPr09
ILPr10
ILPr11
ILPr12
ILPr13
ILPr14
ILPr15
ILPr16
ILPr17
ILPr18
ILPr19
ILPr20
ILPr21
ILPr22
ILPr23
ILPr24
ILPr25
ILPr26
ILPr27
ILPr28
ILPr30
ILPr31
ILPr32
ILPr33
ILPr34
ILPr35
ILPr36
ILPr37
ILPr38
ILPr39
ILPr40
ILPr41
ILPr42
ILPr43
ILPr44
ILPr45
SDPr01
SDPr02
SDPr03
SDPr04
SDPr05
SDPr06
SDPr08
SDPr09
SDPr10
SDPr11
SDPr12
SDPr13
SDPr14
SDPr15
SDPr16
SDPr17
SDPr18
SDPr19
SDPr20
SDPr21
SDPr22
SDPr23
SDPr24
SDPr25
SDPr26
SDPr27
SDPr28
SDPr29
SDPr30
SDPr31
SDPr32
SDPr33
SDPr34
SDPr35
ILPo01
ILPo02
ILPo10
ILPo17
ILPo29
ILPo30
ILPo31
ILPo32
ILPo33
ILPo34
ILPo35
ILPo36
ILPo37
ILPo38
ILPo39
ILPo40
ILPo41
ILPo42
ILPo43
ILPo45
SDPo01
SDPo02
SDPo04
SDPo16
SDPo17
SDPo18
SDPo24
SDPo25
SDPo26
SDPo28
SDPo31
SDPo34
SDPo35
